# Impact of Cognitive Dysfunction in the Middle East Depressed Patients: The ICMED Study

**DOI:** 10.2174/1745017901814010270

**Published:** 2018-10-31

**Authors:** Abdulqader Al Jarad, Ahmad Al Hadi, Ali Al Garatli, Aly Akram, Dakhil Alsaeidi, Fahad Al Mansour, Hany El Amin, Mohamed Khaled, Nawaf Alharthi, Rafat Al Owesie, Samia Abdullah, Talaat Matar, Tarek Darwish

**Affiliations:** 1 Psychiatric Department, King Saud University, King Khalid University Hospital, Riyadh, KSA; 2 Psychiatric Department in King Fahd Medical City, Riyadh, KSA; 3 Psychiatric Department, Cairo University, Egypt and Psychiatric Department at Dr Erfan Hospital, Jeddah, KSA; 4 Psychiatric Departments, King Faisal Specialist Hospital and Research Centre, Jeddah, KSA; 5 Psychiatric Departments, Edrak Medical Center, Riyadh, KSA; 6 Psychiatric Departments, Saudi German Hospital, Jeddah, KSA; 7 Jeddah Mental Health Hospital, Jeddah, KSA; 8 Prince Sultan Military Hospital, Riyadh, KSA; 9 Psychiatric Departments, Rashid Hospital, Dubai Health Authority, UAE; 10 Psychiatric Departments, Saif Hospital, Ras Al-Khaimah, UAE; 11 Psychiatric Departments, Sheikh Khalifa Medical City, Abu Dhabi, UAE

**Keywords:** Depression, Hamilton Depression Rating Scale, IDEA questionnaire, Trail Making Test (TMT)-A and B, Workplace, Cognitive dysfunction

## Abstract

**Background:**

Major depressive disorder is a common condition with a high rate of recurrence, chronicity, and affecting economic burden, including disability in the workplace, which leads to negative consequences on both individuals and society.

**Objectives:**

This study aimed to estimate the impact of cognitive dysfunction, as declared by the patient, on performing daily tasks/activities among patients with major depression disorder (MDD).

**Methods:**

This investigation is based on multinational cross-sectional survey of 499 workers recruited from the Kingdom of Saudi Arabia (KSA) and United Arab Emirates (UAE). We assessed the severity of depression by Hamilton Depression Rating Scale (HDRS). Impact of Depression in the Workplace in Europe Audit (IDEA) survey and trial making test (TMT) parts A and B were used to assess the impact of cognitive dysfunction on performing daily tasks/activities in adult patients presented with MDD.

**Results:**

A total of 499 persons were included in this study, aged 18–66 years, current workers and managers. Of them, 17.8% were normal (remitted), 22.4% were mildly depressed, 23.4% were moderately depressed, 8.6% were severely depressed, and 27.7% were very severely depressed at the time of the study according to HDRS. Common symptoms attributable to depression were low mode or sadness (89.8%), followed by insomnia (75.2%) and crying (70.9%). Of them, low mode or sadness was the most common factor affecting the work performance (90.2%). About 66.3% of participants diagnosed with depression by a doctor/medical professional. Awareness of the disease was recognizable by patients’ managers in only 31.9% of the cases. Furthermore, 45.3% of cases had taken off work due to depression with mean duration of 38.7 (95% CI 37.7 to 39.7) days. The mean TMT parts A and B score were 69.2 (95% CI 66.3 to 72.2) and 126.6 (95% CI 121 to 132), respectively. Lastly, a significant positive correlation between the mean score for HDRS and TMT-A and B scores was observed.

**Conclusion:**

Depression affects work productivity and work environment with negative consequences to countries’ economy. Awareness of depression in the workplace in KSA and UAE is still suboptimal. The personal and societal burden of this issue cannot be neglected when we become aware of the proportion of affected people.

## 1 INTRODUCTION

The American Psychiatric Association defined the Major Depressive Disorder (MDD) as a common and serious medical illness that negatively affects how you feel, the way you think and how you act [1]. MDD is expected to be the second most common cause of disability by 2020 and is expected to account for 15% of the total disease burden [2]. It has been considered a leading cause of disability affecting an estimated 350 million people worldwide [2]. Middle Eastern (ME) countries have a high prevalence of mental disorders, specially MDD, with rates between 5 and 10% [3].

Cognitive dysfunction is defined as deficits in attention, verbal or nonverbal learning, short or long-term memory, visual and auditory processing, processing speed, problem solving, and motor functioning. The cognitive symptoms of depression such as diminished ability to think, concentrate and indecisiveness can have a large impact in the workplace [4].

Depression has a destructive effect on the individual’s ability to work at home, at work, and within everyday social networks. Patients with MDD have compromised productivity due to frequent days off, sick leave, and early retirement with a major impact in the workplace. In addition, depressed patients attempt to make sense of what they are saying and expressing, and they may be unwilling to seek any kind of professional help. These problems can decrease the work performance causing financial loss, further intensifying to become a burden on community [5].

Moreover, absenteeism causes increased workload for other employees in the workplace and decreased output. In addition, reduced productivity at work related to the absenteeism is a major concern for employers [6].

Measurement of cognitive function in depressed patients may enhance our understanding of the association between cognitive function and major depressive disorder. The Impact of Depression in the Workplace in Europe Audit (IDEA) survey was developed by the European depression association in 2012 to measure the impact of depression in the workplace across Europe [7]. IDEA has been previously applied in many countries before (Spain, UK, France, Italy, Germany, Turkey and Denmark) to investigate the impact of depression in the workplace [8, 9].

The aim of this study was to assess the impact of depression on performing daily activities due to cognitive dysfunction in Kingdom of Saudi Arabia (KSA) and United Arab Emirates (UAE) regarding the effect of depression in the workplace, of which currently there is limited information.

## 2 PATIENTS AND METHODS

### 2.1 Study Design

The current multinational cross-sectional study – the ICMED study (Impact of Cognitive Dysfunction in the Middle East Depressed Patients) - was conducted between May and December 2016 in the whole KSA and UAE and was included patients with MDD without any follow up period.

### 2.2 Study Population

Patients were recruited from psychiatry outpatient offices in KSA and UAE. Site selection was done to have a real representation in both KSA and UAE considering the diverse population, geographical distribution, and category of practice including private, institutional and governmental centers. We include the patients who 1) aged 18-60 years; 2) had a MDD, characterized by symptoms of daily depression at least for a period of two weeks and with a previous history of maximum three past depressive episodes as per the psychiatrist’s assessment with a maximum of two antidepressants treatments at the time of the study; and 3) who were full time, part time working or currently not working but were previously employed. Exclusion criteria were: 1) patients having a psychotic manifestation; 2) patients having an associated axis I psychiatric illness; 3) patients with severe or unstable medical condition; 4) patients who current diagnosis of substance abuse; 5) patients who were pregnancy or lactation; 6) patients how was treated by antipsychotic and/or benzodiazepine 7) patients who exposed to electroconvulsive therapy in the last 6 months; 8) patients who were not able to perform Trail Making Test (TMT) parts A and B; 9) patients not able to read and comprehend properly as per physician assessment; and 10) patients who were participating or had participated in an interventional trial. To avoid any source of potential bias, patients who met the inclusion criteria were included consecutively in the study.

### 2.3 Data Collection and Measures

Data were recorded by the physician in an electronic Case Report Form. We collected the following data from each patients: (1) socio-demographic characteristics, (2) family history of mental illnesses, (3) concomitant medical illnesses (4) date of diagnosis of major depression, (5) current disease severity assessed by the investigator through the Hamilton Depression Rating Scale, 17-items (HDRS), (6) assessment the remission of depression as per the investigator judgment assessment, (7) previous and current medical treatments for depression and other treatments and (8) the physician or the nurse filled the IDEA and TYT parts A and B questionnaires from each patients. Depression currently in remission was assessed by the investigator judgment. All serious and non-serious Adverse Drug Reactions (ADRs) spontaneously reported by the patient or observed by the investigator related to any drug or study procedure were reported according to local regulations.

#### 2.3.1 Hamilton Depression Rating Scale (HDRS)

This questionnaire was designed to be used by a health care professional during a clinical interview with an already identified depressed patient. It consists of 17 items that measure the severity of depressive symptoms and as examples, the interviewer rates the level of agitation clinically noted during the interview. Scoring was based on the 17-item scale and scores of 0-7 are considered as being normal, 8-13 suggest mild depression, 14-18 moderate depression, 19-22 severe depression and scores over 23 are indicative of severe depression [10, 11].

#### 2.3.2 The Impact of Depression in the Workplace in Europe Audit (IDEA) Survey

IDEA survey (a 13 items questionnaire) was elaborated by members of the European Association of Depression [7]. All participants have to score health conditions from the least disabling (1) to the most disabling one (5) according to five health problems heart/circulation problem/blood pressure; hearing loss/deafness; depression; alcoholism/alcohol abuse; and cerebrovascular disease. Then, the participants were asked if they were diagnosed with depression by a doctor or medical professional.

The question “have you ever taken time off work because of your depression?” was asked to every ever-depressed worker. All workers reported if there were any colleagues at their workplace that ever had depression, how they came to know about the depression of the colleague, and what they did about this. If the respondent was a manager, they also answered on available resources to support depressed employees and how convenient was the support.

#### 2.3.3 Trail Making Test (TMT) Parts A and B

Both parts of the Trail Making Test consist of 25 circles distributed over a sheet of paper. In Part A, the circles were numbered 1-25, and the patient should draw lines to connect the numbers in ascending order. In Part B, the circles included both numbers (1-13) and letters (A-L); as in Part A, the patient drew lines to connect the circles in an ascending pattern, but with the added task of alternating between the numbers and letters (*i.e.*, 1-A-2-B-3-C, *etc*.). The patient should connect the circles as quickly as possible, without lifting the pen or pencil from the paper. When an error was made, the participant was instructed to return to the “circle” where the error originated and continue. Time to complete each part was recorded [12, 13].

### 2.4 Statistical Methodology

A convenience sample was taken of adult MDD patients consecutively admitted at the study sites. All those admitted between May and December 2016 were included. Patients’ data were considered valuable if they answered the two parts of the TMT and at least one question in the IDEA questionnaire.

Continuous variables were assessed for normal distribution using the Kolmogorov-Smirnov statistical test. For normally distributed data, we used mean and confidence interval (95% CI). While for not-normally distributed data, we used median and rage. Categorical variables were presented as frequencies and percentages. Pearson Chi-Square tests were used to determine the association between the categorical variables. Independent t-test was used to assess the difference between any continues variables. Correlation analysis was used when required. All data were analyzed using SAS statistical software, version 9.3 (SAS Institute Inc., Cary, NC).

### 2.5 Ethical Considerations

Eligible patients were only included in the study after providing written Institutional Ethics Review Boards (IRB)-approved informed consent. This study was also designed, implemented and reported in accordance with the Guidelines for Good Pharmacoepidemiology Practices (GPP) of the International Society for Pharmacoepidemiology (ISPE 2007), the STROBE guidelines [14], and with the ethical principles laid down in the Declaration of Helsinki. Ethics committees’ approvals were collected prior to performing the study. The following ethics committees approved on the conduction of the study; *Dubai Scientific Research Ethics Committee***-**Dubai Health Authority, *Sheikh Khalifa Medical City Institutional Review board-*Research Ethics Committee, *Ministry of health*, Research Ethics Committee, *Al Ain Hospital Research Ethics Committee*, *King Saud University Medical City Institutional Review Board*, *Research and Ethics Committee*-Sultan Bin Abdulaziz-Humanitarian City, *King Fahad Medical City Institutional Review Board*, and *KFSHR Institutional Review Board*.

## 3 RESULTS

### 3.1 Description of the Overall Study Population

Data were collected from 499 eligible subjects from 01 May 2016 to 31 December 2016 in KSA and UAE, Fig. (**1**). Of them, 89 (17.8%) patients were normal (remitted), 112 (22.4%) had mild depression, 117 (23.4%) had moderate depression, 43 (8.6%) severe depression, and 138 (27.7%) were severely depressed according to HDRS. Table **1** presents the patients’ demographics classified according to the severity of depression. The majority of respondents with or without an experience of depression was Male (57%), aged 25 to 34 years (37%), married (62%), with high education below the university degree (44%), and working full-time (77%). Almost half of the patients (45.7%, n=228) work in large companies (>250 employees).

### 3.2 Description of the Main Section of the IDEA Questionnaire

#### 3.2.1 Items Related to Health Problems and Diagnosis of Depression in the Overall Population

Using IDEA questionnaire, the majority of the patients (95.6%, 475) were experiencing depression, followed by burn-out (89.5%, n=445), Fig. (**2**). In parallel, 331 (66.3%) patients declared that they have been personally diagnosed as having depression by a doctor or medical professional, while 321 patients (64.3%) declared that they know someone who has been diagnosed with depression by a doctor.

In addition, if patients ever diagnosed with depression, 340 (68.1%) patients would tell their partner or family about their diagnosis while 40 (8.0%) patients did not know if they would do it.

Using a 1 (the least disabling) to 5 (the most disabling) scale, the mean scores of the five health conditions that have a detrimental impact on a person's ability to perform normal day to day activities in the 499 patients were analyzed. The mean of cerebrovascular diseases was the higher impact on patient’s day activity 4.1 (95% CI 3.99 to 4.21), followed by depression 3.8 (95% CI 3.69 to 3.9), alcohol use disorders 3.4 (95% CI 3.28 to 3.52), hearing disorder 2.8 (95% CI 2.69 to 2.91), and cardiovascular disorders 2.5 (95% CI 2.39 to 2.61).

#### 3.2.2 Items Related to Work in the Overall Population

**Fig. (3):**
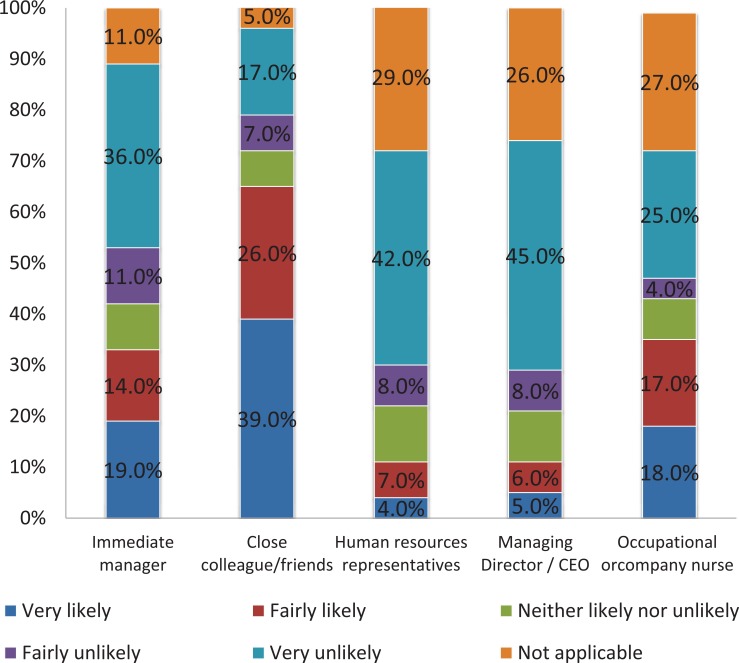
Likelihood to tell others about one’s depression.

Regarding the depressed patients: 222 (44.6%) of them declared that they would not tell their employer about their diagnosis. The main reason for not disclosing the truth was privacy as the patients would not want to tell anyone about their condition (36%, n=182). In parallel, 238 (47.8%) patients had never personally taken any time off work because of their depression. Three-quarters of the patients (76%, n=378) declared they did not require a career the last time they were off work due to depression. On average, 494 patients took 38.7 (95% CI 37.7 to 39.7) days off work during their last episode of depression. Results about the likelihood for the patients to tell other people about their depression are displayed in (Fig. **3**).

#### 3.2.3 Items Related to Symptoms and Health Status in the Overall Population

Among the attributes/symptoms that had impacted on the ability to perform tasks at work, the most troublesome were: Low mood or sadness (89.8%, n=448), followed by sleeping problems and insomnia (75.2%, n=375). Low mood or sadness was also the most common issue that would have the most impact on the patients’ availability to perform tasks at work as normal (90.2%, n=450) and that would be most likely to cause them to take time off from work (89.4%, n=446), followed by concentration problems (81.6%, n=407 and 79.8%, n=398, respectively). These two symptoms were also the most frequent ones which would encourage the patients to return to work when improved: low mood or sadness (86.4%, n=431) and concentration problems (76.2%, n=380), Table **2**. As for the behaviors declared by the patients as signs that someone in the workplace is depressed, withdrawal from colleagues was the most frequent one (80.0%, n=399), followed by crying at work (62.5%, n=312), increased or prolonged sick leave (52.5%, n=262) and making more mistakes than usual (49.7%, n=248).

#### 3.2.4 Items Answered from an Employer Perspective in the Overall Population

A total of 196 (39.3%) employers assumed they know that one more employee had depression while working for them, 85 (17.0%) employers said they think that one more employee had depression while working for them, while 71 (14.2%) employers declared that none of their employees had ever had depression and 147 (29.5%) employers said they do not know. These employers knew about their employees’ depression mainly based on their own suspicion (31.9%, n=159), and 150 (30.1%) employers knew about it from the employees themselves. Then, the employers mainly encouraged the employees to talk to a healthcare professional (31.1%, n=155), 86 (17.2%) employers discussed with them and asked if there was anything they could do to help, while 113 (22.6%) did nothing. The employers declared that their employees take an average of 25.8 days off work by year due to depression. To deal with the employees’ depression, 246 (49.3%) employers declared that they can provide a support from a medical professional while 138 (27.7%) said they have no formal support or resources in place. Overall, this support enabled very well 150 (30.1%) employers to deal with employees who have/had depression while it was not at all well for 91 (18.2%) employers. The most common ways declared by the employers to be useful to support employees with depression at the workplace were counselors or counseling services (49.7%, n=248) and better government legislation/policies to protect employees (40.5%, n=202).

#### 3.2.5 Items Related to Health Problems and Diagnosis According to the Severity of Depression

Using a 1 to 5 scale, from the least disabling (1) to the most disabling one (5), the mean (95% CI) scores of the five health problems in the currently normal patients were, by ascending order: 2.7 (2.45 to 2.95) for heart/blood pressure or circulation problems, 2.7 (2.43 to 2.97) for hearing loss/deafness, 3.3 (3.05 to 3.55) for depression, 3.3 (3.01 to 3.59) for alcohol use disorders, and 3.6 (3.29 to 3.91) for cerebrovascular disease. The same tendency was observed in patients with mild, moderate, severe and very severe depression Table **3**.

Using IDEA questionnaire, the majority of the normal patients (96.6%, 86), patients with mild depression (90.1%, n=100), moderate depression (95.7%, n=112), severe depression (95.2%, n=40) and very severe depression (99.3%, n=137) said they are experiencing depression, followed by burn-out and stress. In parallel, 47 (52.8%) normal patients declared they have not been personally diagnosed as having depression by a doctor/medical professional. In parallel, more than the half of patients with mild depression (66.1%, n=74), moderate depression (67.5%, n=79), severe depression (79.1%, n=34) and very severe depression (73.9%, n=102) declared the opposite. The same tendency is observed with regards knowing someone who had been diagnosed with depression by a doctor/medical professional. Also, if they ever diagnosed with depression, more than the half of patients would tell their partner/family about their diagnosis: 55 (61.8%) normal patients, 74 (66.1%) patients with mild depression, 75 (64.1%) patients with moderate depression, 23 (53.5%) patients with severe depression and 113 (81.9%) patients with very severe depression.

#### 3.2.6 Items Related to Work According to the Severity of Depression

If ever diagnosed with depression, almost half of the normal patients and patients with mild, moderate and severe depression declared they would not tell their employer about their diagnosis, while 65 (47.1%) patients with very severe depression declared they would. The main reason for not disclosing the truth was privacy as the patients would not want to tell anyone about their condition: normal patients (42%, n=37), mild depression (42%, n=47), moderate depression (38%, n=44), severe depression (42%, n=18) and very severe depression (26%, n=36). In addition, almost half of the normal patients and patients with mild, moderate and severe depression declared they had never personally taken any time off work because of their depression while 78 (56.5%) patients with very severe depression did take days off work. Almost three-quarters of the patients of all depression categories declared they did not require a carer the last time they were off work due to depression.

Results about the likelihood for the patients to tell other people about their depression by severity of their disease are displayed in Table **4**.

#### 3.2.7 Items Related to Symptoms and Health Status According to the Severity of Depression

Low mood or sadness was the most common attribute associated by the patients with depression, regardless of the disease severity: Normal patients (79.8%, n=71), mild depression (86.6%, n=97), moderate depression (91.5%, n=107), severe depression (90.7%, n=39) and very severe depression (97.1%, n=134). Low mood or sadness were the most common issues that would have the most impact on the patients’ availability to perform tasks at work as normal for all types of depression [mild depression: 99 (88.4%) patients; moderate depression: 111 (94.9%) patients; severe depression: 40 (93.0%) patients and very severe depression: 129 (93.5%) patients], except for normal patients who mainly chose concentration problems (82.0%, n=73). Low mood or sadness were also the most common issues that would be most likely to cause them to take time off from work, regardless of the disease severity: Normal patients (84.3%, n=75), mild depression (82.1%, n=92), moderate depression (90.6%, n=106), severe depression (93.0%, n=40) and very severe depression (96.4%, n=133). These two symptoms (low mood/sadness) were also the most frequent ones which would encourage the patients to return to work when improved, regardless of the disease severity: Normal patients (69.7%, n=62), mild depression (84.8%, n=95), moderate depression (91.5%, n=107), severe depression (95.3%, n=41) and very severe depression (91.3%, n=126). As for the behaviors declared by the patients as signs that someone in the workplace is depressed, withdrawal from colleagues was the most frequent one, followed by crying at work, increased or prolonged sick leave and making more mistakes than usual.

#### 3.2.8 Items Answered from an Employer Perspective According to the Severity of Depression

In the group of normal persons, 16 (18.0%) employers assumed they know that one more employee had depression while working for them, 9 (10.1%) employers said they think that one more employee had depression while working for them, while 23 (25.8%) employers declared that none of their employed had ever depression and 41 (46.1%) employers said they do not know. The same tendency is observed with the employers of patients with mild, moderate, severe and very severe depression. These employers knew about their employees’ depression mainly because they suspected the patient had depression [normal patients: 31 (34.8%) patients; mild depression: 45 (40.2%) patients; moderate depression: 45 (38.5%) patients; severe depression: 21 (48.8%) patients and very severe depression: 17 (12.3%) patients]. Then, the employers mainly encouraged the employees to talk to a healthcare professional and asked if there was anything they could do to help. The employers declared that their employees take an average of 18.9 days (range, 0 to 150) off work by year due to depression (normal patients), 26.3 days (range 0 to 120) off work for mild depression, 17.4 days (range 2 to 30) off work for moderate depression, 16.1 ± 21.6 days (range 3 to 60) off work for severe depression, 32.5 days (for 7 to 60) off work for very severe depression. The most common way declared by the employers to be useful to support employees with depression at workplace were counselors or counseling services [normal patients: 37 (41.6%) patients; mild depression: 48 (42.9%) patients; moderate depression: 41 (35.0%) patients; severe depression: 18 (41.9%) patients and very severe depression: 104 (75.4%) patients].

### 3.3 Cognitive Dysfunction Assessed by the TMT Parts A and B Tests

The mean TMT part A score was 69.2 (95% CI 66.3 to 72.2). For TMT part B, the mean score was 126.6 (95% CI 121 to 132). In TMT-part A, 67.6% patients had a normal score (≤78 seconds) while 155 (32.4%) patients had a deficient score (>78 seconds). Also, 97.0% patients had a normal score (≤273 seconds) while 14 (3.0%) patients had a deficient score (>273 seconds) in TMT-part B. Detailed data according to the severity of depression were reported in Table **5**. We compared the mean TMT score parts A and B between the patients with impairment in productivity and patients without impairment in productivity. We revealed a statistically significant increase in both TMT score A and B in patients with impairment in productivity (Mean difference= 6.6, 95% CI (0.47 to 12.71), p=0.003 for TMT-A and 19.6, 95% CI (9.01 to 30.2), p<0.001. This result was supported by Spearman's rho correlation analysis which revealed a statistically significant positive correlation between TMT scores and impairment in productivity (r=0.21, p=0.006 for TMT-A and r=0.38, p<0.001 for TMT-B). Furthermore, Our results showed a statistically significant association between the degree of cognitive impairment and work dysfunction in the included patients (Chi^2^= 4.11, P=0.04).

### 3.4 Hamilton Depression Rating Scale (HDRS)

The mean total score for HDRS (17-items) was 15.8 (95% CI 15.1 to 16.5). Regarding the first item of the scale (depressed mood), most of the 499 patients (46.3%, n=231) stated that their mood is spontaneously and verbally. As for the feelings of guilt, 236 patients (47.3%) indicated they felt they let people down. In addition, 314 (62.9%) patients had no suicidal thoughts.

**Fig. (4):**
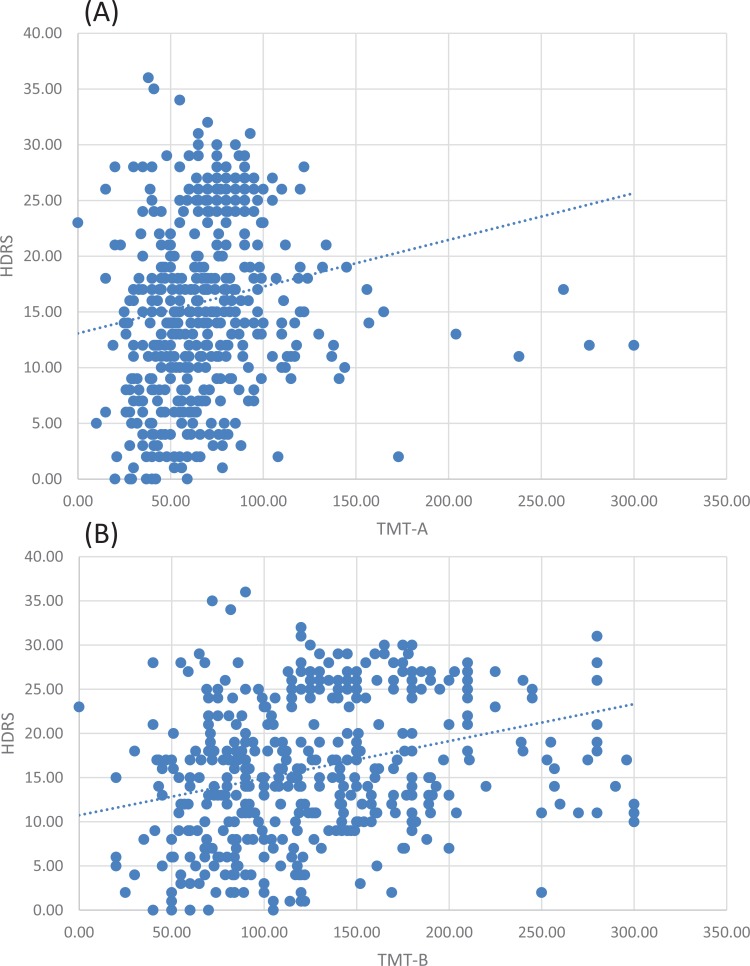
Correlation between the Hamilton Rating Scale and Trial Making Tests.

Our study reported a significant positive correlation between the mean score for HDRS and TMT-A score (r=0.31, p<0,0001) and TMT-B score (r=0.42, p<0,0001), (Fig. **4**).

## 4 DISCUSSION

This cross-sectional and multinational non-interventional study aimed to estimate the impact as declared by the patient, of cognitive dysfunction on performing daily tasks/activities among patients with depression in KSA and UAE using IDEA questionnaire and TMT parts A and B tests. It also aimed to describe cognitive dysfunction by the time taken to perform TMT parts A and B in remitted and non-remitted depressed patients, according to the number of depressive episodes and severity as assessed by the investigator using the HDRS. The time allocated to enroll 500 patients was 5 months. However, data were collected from 01 May 2016 until 31 December 2016 for analysis and the study population consisted of 499 eligible patients.

Most of the included patients were moderately depressed. Almost three-quarters of the included patients had a full-time employment and most of them were married. Furthermore, the study reported that patients with more severe depression reported more impairment in productivity than those with less severe depression. This confirms previous findings by international studies conducted in Brazil [15], [16], Australia, Spain, Russian Federation and USA [16] in primary care settings, and in Brazil as well among 1,000 workers recruited from online sources [17].

Less than half of the participants (39.3%) were not able to recognize probably depressed workers and the awareness was more frequently associated to the manager's suspicion (31.9%) and self-disclosure by the depressed worker (30.1%). Helpful and supportive behaviors were frequently observed among 49.3% of the managers whereas 27.7% employers showed no helping attitude. The quality of support received from the company to handle depressed employees was evaluated as very good by the majority of employers managing mildly to severely depressed employees. It was mainly evaluated as not at all well by the majority of employers in case of very severe depression. These findings are in line with the literature in relation to the employers' perspective [17]. They also suggest a window of opportunity for preventive actions, such as early identification, proper referral, and adequate resource provision, since some managers and workers were not capable of recognizing and helping persons suffering from depressive symptoms.

Some cautions should be born in mind in the data interpretation before generalizing the findings to the workforce in KSA and UAE. First, some of the data collected were based on self/patient-reported outcomes, which are limited by social desirability [18] and recall bias. Social desirability is a phenomenon which restricts the extent to which the study participants will ‘speak their minds’. It is associated with the participants’ predisposition to give more socially acceptable answers to a questionnaire, and the fact that people don’t always ‘know their minds’ [19]. In addition, the probability of recall bias cannot be excluded because the employees self-reported subjective experience of depression in the past, producing distortions in the accuracy or completeness of the retrieved recollections. The second limitation is that many variables that were supposed to be analyzed were not available such as family history of mental illnesses, time since diagnosis of major depression, number of episodes since diagnosis, previous treatments for depression received by study population. Thus, data analysis according to the number of depressive episodes was not done contrarily to what was planned in the statistical analysis plan.

## CONCLUSION

In conclusion, the present study might be the first footstep of a large national campaign to fight against depression in currently or previously employed patients in KSA and UAE. It also draws attention to the negative consequences of depression in currently or previously employed persons, since it affects many domains such as work productivity, environment climate, with negative consequences to countries’ economy. The personal and societal burden of this issue cannot be neglected when we become aware of the proportion of affected people.

## Figures and Tables

**Fig. (1):**
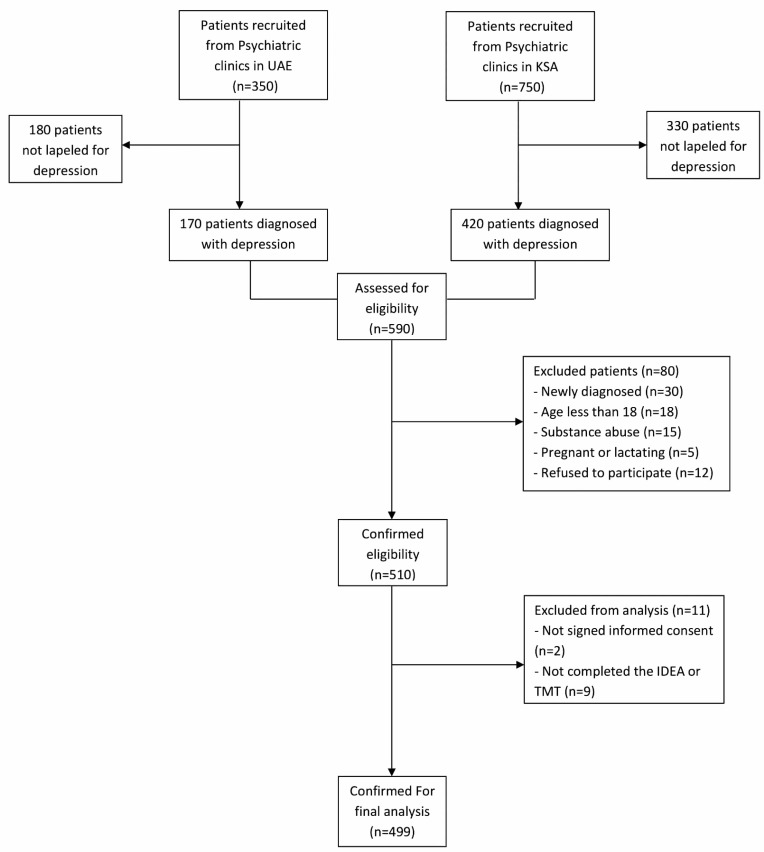
Flow diagram of study participants.

**Fig. (2):**
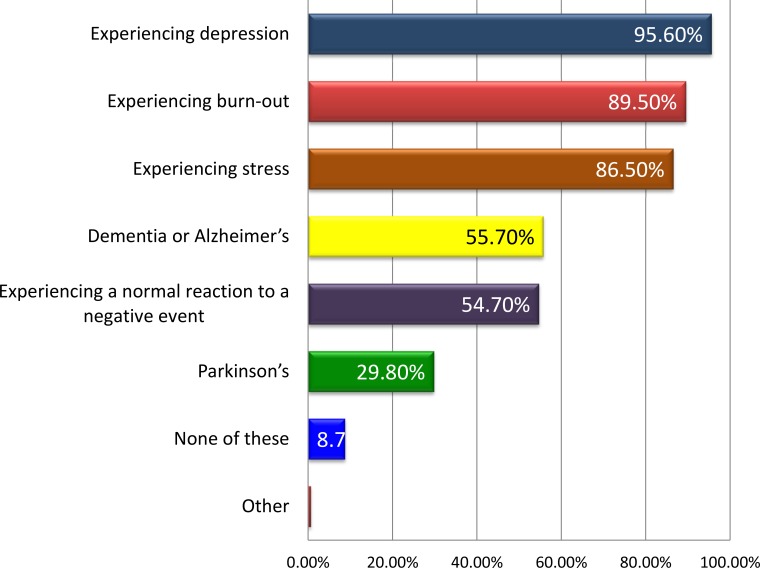
Suspected problem in patients suffering several issues.

**Table 1:** Socio-demographic characteristics of the patients by severity of depression.

	**Normal** **(n=89)**		**Mild** **(n=112)**		**Moderate ** **(n=117)**		**Severe** **(n=43)**		**Very severe** **(n=138)**		**Over All** **(n=499)**	
Gender												
Male	34	38.2%	54	48.2%	62	53.0%	26	60.5%	109	79.0%	285	57.1%
Female	55	61.8%	58	51.8%	55	47.0%	17	39.5%	29	21.0%	214	42.9%
Age group												
18-24 years	10	11.2%	8	7.1%	7	6.0%	4	9.3%	9	6.5%	38	7.6%
25-34 years	34	38.2%	42	37.5%	51	43.6%	15	34.9%	46	33.3%	188	37.7%
35-44 years	27	30.3%	40	35.7%	39	33.3%	14	32.6%	46	33.3%	166	33.3%
45-54 years	14	15.7%	9	8.0%	13	11.1%	6	14.0%	31	22.5%	73	14.6%
55-59 years	2	2.2%	7	6.3%	6	5.1%	3	7.0%	6	4.3%	24	4.8%
60 years	2	2.2%	6	5.4%	1	0.9%	1	2.3%	0	0.0%	10	2.0%
Prefer not to say	0	0.0%	0	0.0%	0	0.0%	0	0.0%	0	0.0%	0	0.0%
Type of employment												
Full-time employment	59	66.3%	73	65.2%	90	76.9%	31	72.1%	127	92.0%	380	76.2%
Part-time employment	6	6.7%	13	11.6%	12	10.3%	6	14.0%	4	2.9%	41	8.2%
Previously employed in the last 12 months	5	5.6%	8	7.1%	5	4.3%	4	9.3%	6	4.3%	28	5.6%
Not working	17	19.1%	18	16.1%	10	8.5%	2	4.7%	1	0.7%	48	9.6%
Prefer not to say	2	2.2%	0	0.0%	0	0.0%	0	0.0%	0	0.0%	2	0.4%
Level of education												
Secondary school or earlier	22	24.7%	27	24.1%	27	23.1%	10	23.3%	44	31.9%	130	26.1%
Professional qualification of degree standard or above	11	12.4%	16	14.3%	26	22.2%	11	25.6%	39	28.3%	103	20.6%
Higher education below university degree level	44	49.4%	56	50.0%	55	47.0%	18	41.9%	46	33.3%	219	43.9%
University degree (including polytechnic or college degree)	12	13.5%	13	11.6%	9	7.7%	4	9.3%	9	6.5%	47	9.4%
Marital status												
Married	47	52.8%	66	58.9%	73	62.4%	23	53.5%	100	72.5%	309	61.9%
Bachelor	32	36.0%	29	25.9%	29	24.8%	18	41.9%	30	21.7%	138	27.7%
Widowed	0	0.0%	5	4.5%	2	1.7%	0	0.0%	1	0.7%	8	1.6%
Divorced	8	9.0%	10	8.9%	7	6.0%	1	2.3%	4	2.9%	30	6.0%
Separated	1	1.1%	1	0.9%	4	3.4%	0	0.0%	2	1.4%	8	1.6%
Do not know	0	0.0%	0	0.0%	0	0.0%	0	0.0%	0	0.0%	0	0.0%
Prefer not to say	1	1.1%	1	0.9%	2	1.7%	1	2.3%	1	0.7%	6	1.2%

**Table 2:** Description of the main section of the IDEA questionnaire (items related to symptoms and health status) (n=499).

	**Symptoms Indicating Depression**		**Symptoms Impacting the Work Performance**		**Symptoms Causing Taken off Work**		**Improvement Symptoms that Encourage you to Return to Work**	
Low mood or sadness	448	89.8%	450	90.2%	446	89.4%	431	86.4%
Concentration problems	328	65.7%	407	81.6%	398	79.8%	380	76.2%
Crying for no reason	354	70.9%	180	36.1%	221	44.3%	183	36.7%
Indecisiveness	240	48.1%	205	41.1%	168	33.7%	155	31.1%
Forgetfulness	250	50.1%	248	49.7%	198	39.7%	204	40.9%
Difficulty planning day to day activities	265	53.1%	217	43.5%	242	48.5%	214	42.9%
Changes in weight and appetite	308	61.7%	135	27.1%	107	21.4%	88	17.6%
Sleeping problems/insomnia	375	75.2%	372	74.5%	390	78.2%	344	68.9%
Loss of interest in daily activities	343	68.7%	287	57.5%	306	61.3%	192	38.5%
Other symptoms	20	4.0%	0	0.0%	0	0.0%	0	0.0%

**Table 3:** Impact of health problems on patients by severity of depression.

	**Normal** **(n=89)**	**Mild depression** **(n=112)**	**Moderate depression** **(n=117)**	**Severe depression** **(n=43)**	**Very severe depression** **(n=138)**
Heart, blood pressure or circulation problems Mean (95% CI)	2.7 (2.45 - 2.95)	2.6 (2.36 - 2.84)	2.8 (2.56 - 3.04)	2.8 (2.47 - 3.13)	1.8 (1.65 - 1.95)
Hearing loss, adult onset. Mean (95% CI)	2.7 (2.43 - 2.97)	3 (2.74 - 3.26)	3 (2.76 - 3.24)	3.2 (2.81 - 3.59)	2.4 (2.25 - 2.55)
Depression Mean (95% CI)	3.3 (3.05 - 3.55)	3.6 (3.38 - 3.82)	3.8 (3.6 - 4)	3.8 (3.44 - 4.16)	4.3 (4.15 - 4.45)
Alcohol use disorders Mean (95% CI)	3.3 (3.01 - 3.59)	3.4 (3.12 - 3.68)	3.4 (3.13 - 3.67)	3.1 (2.68 - 3.52)	3.9 (3.73 - 4.07)
Cardiovascular disease Mean (95% CI)	3.6 (3.29 - 3.91)	4 (3.74 - 4.26)	3.9 (3.66 - 4.14)	3.8 (3.44 - 4.16)	4.6 (4.45 - 4.75)

CI: Confidence interval

**Table 4:** Likelihood to tell others about one’s depression by severity of depression.

-	**Normal** **(n=89)**	**Mild** **(n=112)**	**Moderate** **(n=117)**	**Severe** **(n=43)**	**Very severe** **(n=138)**
Immediate manager					
Very likely	16%	14%	12%	9%	35%
Fairly likely	11%	15%	11%	23%	13%
Neither likely nor unlikely	11%	7%	9%	7%	9%
Fairly unlikely	7%	9%	8%	21%	16%
Very unlikely	42%	37%	55%	28%	19%
Not applicable	13%	18%	4%	12%	9%
Close colleague/friends					
Very likely	36%	33%	34%	33%	50%
Fairly likely	25%	22%	29%	14%	31%
Neither likely nor unlikely	8%	7%	5%	5%	9%
Fairly unlikely	4%	8%	7%	21%	2%
Very unlikely	20%	22%	22%	21%	5%
Not applicable	7%	7%	2%	7%	3%
Human Resources representative					
Very likely	9%	3%	4%	5%	1%
Fairly likely	6%	7%	8%	7%	8%
Neither likely nor unlikely	7%	11%	11%	7%	14%
Fairly unlikely	10%	4%	7%	14%	8%
Very unlikely	53%	45%	59%	51%	16%
Not applicable	16%	30%	11%	16%	54%
Managing Director					
Very likely	6%	7%	8%	7%	1%
Fairly likely	6%	9%	9%	9%	1%
Neither likely nor unlikely	8%	5%	8%	5%	19%
Fairly unlikely	7%	5%	8%	16%	9%
Very unlikely	58%	48%	58%	44%	22%
Not applicable	16%	25%	10%	19%	47%
Occupational or Company nurse					
Very likely	18%	21%	13%	7%	24%
Fairly likely	18%	13%	24%	14%	15%
Neither likely nor unlikely	8%	10%	11%	5%	5%
Fairly unlikely	4%	3%	3%	21%	2%
Very unlikely	29%	29%	32%	35%	11%
Not applicable	22%	23%	17%	19%	43%

**Table 5:** Description of TMT parts A and B according to the severity of depression.

		**Normal** ** (n=89)**	**Mild** **(n=112)**	**Moderate ** **(n=117)**	**Severe** ** (n=43)**	**Very severe** ** (n=138)**
TMT part A score. Mean (95% CI)		53.3 (48.2 - 58.4)	73.4 (64.7 - 82.1)	69.8 (63.5 - 76)	70.6 (61.2 -80)	74.6 (71.3 - 77.9)
TMT part B score. Mean (95% CI)		89.2 (80 - 98.4)41.0	128.1 (117 - 139)	123.8 (113 - 135)	134.1 (113 - 55)	147.3 (139 - 156)
Categorized score in TMT part A	Normal (≤78 seconds)	74 (89.2%)	74 (68.5%)	78 (69.0%)	27 (69.2%)	70 (51.9%)
	Deficient (>78 seconds)	9 (10.8%)	34 (31.5%)	35 (31.0%)	12 (30.8%)	65 (48.1%)
Categorized score in TMT part B	Normal (≤78 seconds)	76 (100%)	103 (96.3%)	104 (96.3%)	36 (92.3%)	127 (97.7%)
	Deficient (>78 seconds)	0	4 (3.7%)	4 (3.7%)	3 (7.7%)	3 (2.3%)

## LIST OF ABBREVIATIONS

CEO: = Chief Executive Officer,
e-CRF: = electronic Case Report Form,
ICMED: = Impact of Cognitive Dysfunction in Middle East,
IDEA: = Impact of Depression in the workplace in Europe Audit,
IRB: = Institutional Review Boards,
GPP: = Good Pharmacoepidemiology Practices,
KSA: = Kingdom of Saudi Arabia, Max: Maximum,
ME: = Middle East,
STROBE: = Strengthening The Reporting of Observational Studies,
TMT: = Trail Making Test, UAE: United Arab of Emirates.
